# A High Optical Throughput Spectral Imaging Technique Using Broadband Filters

**DOI:** 10.3390/s20164387

**Published:** 2020-08-06

**Authors:** Duo Wang, Zhe Chen, Xingxiang Zhang, Tianjiao Fu, Rui OuYang, Guoling Bi, Longxu Jin, Xiaoxu Wang

**Affiliations:** 1 Institute of Space Sciences, Institute of Frontier and Interdisciplinary Science, Shandong University, Shandong 266237, China; 2 Changchun Institute of Optics, Fine Mechanics and Physics, Chinese Academy of Sciences, Changchun 130033, China; wangduo161@mails.ucas.ac.cn (D.W.); chenzhe@ciomp.ac.cn (Z.C.); zhangxx@ciomp.ac.cn (X.Z.); futianjiao@ciomp.ac.cn (T.F.); ouyangrui16@mails.ucas.ac.cn (R.O.); biguoling@ciomp.ac.cn (G.B.); Jinlx@ciomp.ac.cn (L.J.); 3 College of Materials Science and Opto-Electronic Technology, University of Chinese Academy of Sciences, Beijing 100049, China

**Keywords:** multi-channel measurement, Tikhonov regularization, reconstructed spectra, overdetermined equations, panchromatic imaging

## Abstract

To address the miniaturization of the spectral imaging system required by a mounted platform and to overcome the low luminous flux caused by current spectroscopic technology, we propose a method for the multichannel measurement of spectra using a broadband filter in this work. The broadband filter is placed in front of a lens, and the spectral absorption characteristics of the broadband filter are used to achieve the modulation of the incident spectrum of the detection target and to establish a mathematical model for the detection of the target. The spectral and spatial information of the target can be obtained by acquiring data using a push-broom method and reconstructing the spectrum using the GCV-based Tikhonov regularization algorithm. In this work, we compare the accuracy of the reconstructed spectra using the least-squares method and the Tikhonov algorithm based on the L-curve. The effect of errors in the spectral modulation function on the accuracy of the reconstructed spectra is analyzed. We also analyze the effect of the number of overdetermined equations on the accuracy of the reconstructed spectra and consider the effect of detector noise on the spectral recovery. A comparison between the known data cubes and our simulation results shows that the spectral image quality based on broadband filter reduction is better, which validates the feasibility of the method. The proposed method of combining broadband filter-based spectroscopy with a panchromatic imaging process for measurement modulation rather than spectroscopic modulation provides a new approach to spectral imaging.

## 1. Introduction

**Figure 1:**
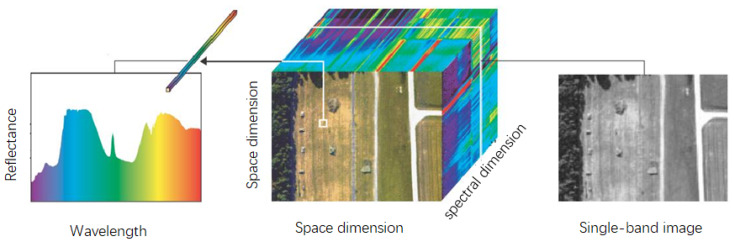
Three-dimensional structure of hyperspectral image.

Hyperspectral remote sensing is a technique used for the continuous remote sensing imaging of ground objects with a very narrow and continuous spectral channel. It is developed based on imaging and spectroscopy. Simultaneously imaging the target in tens to hundreds of wavebands, each band forms a two-dimensional space image, and a three-dimensional hyperspectral image (data) cube can be formed by superimposing multiple two-dimensional space images in spectral dimensions (as shown in Figure 1). Hyperspectral remote sensing technology has the unique advantages of high spectral resolution and spectrum unity, representing a revolutionary step in the history of remote sensing technology development [1,2,3]. Based on this 3D spectral data cube, which can acquire both 2D spatial and 1D spectral information, it is possible to detect and identify targets that are difficult to detect and identify with conventional imaging techniques [4,5,6,7,8,9,10].

Spectral imaging data include 2D spatial information and 1D spectral information, whereas imaging detectors are 2D detectors and can only acquire 2D information at a single transient. Therefore, there are usually two ways to obtain 3D spectral data: (1) obtain 2D spatial information in a narrow band at a time, such as the filter wheel switching method or acousto-optic, liquid crystal and other tunable filter imaging methods; (2) obtain 1D spatial information plus 1D spectral information, such as spectral imaging with slits. According to the different spectral spectroscopic methods, spectral imaging techniques are mainly divided into dispersive, filtered and interferometric methods. Conventional spectral imaging techniques are mainly dispersive [11,12,13] and are typically characterized by a slit aperture in the primary image, which significantly reduces the luminous flux of the system, resulting in a significant limitation in the application of the spectral detection of faint targets. The traditional filtered spectral imaging technique adds a switching mechanism with narrow-band filters into the wideband imaging optical path; one narrow-band filter cuts into the optical path every time and obtains the narrow-band spatial image of the band. The currently used liquid crystal tunable filter (LCTF) is made of liquid crystal with an electronically controlled birefringence effect and is a new type of spectroscopic device [14] which adjusts the phase difference caused by birefringent liquid crystal by applying different voltages so that light of different wavelengths interacts and achieves the continuous tunable scanning of different wavelengths. However, LCTF adopts a polarizer for polarization and bias detection, which makes the light energy utilization low, and the detector needs to use a low-light wideband detector or an intensifier, which is not conducive to target detection and identification and limits the practical application. For an interferometric imaging spectrometer [15,16,17,18,19], a time-modulated fast Fourier transform (FFT) spectroscopic imager using a Michelson interferometer as the spectroscopic element can only obtain an interferogram of a 2D scene with one optical range difference at a time, relying on the scanning motion of a moving mirror to produce an interferogram with a different optical range. There is a need for accurate position scanning due to the reliance on moving mirrors to change the optical range, and a large amount of moving mirror movement can increase the size of the system when an increased spectral resolution is required. The spatially modulated spectral imager has a slit, which reduces the luminous flux of incident light and reduces the signal-to-noise ratio [20,21].

To solve the problems of traditional spectral imaging, new types of computational spectral imaging technologies such as computational tomography [22], the Hadamard transform [23] and compression coding [24,25,26] have been gradually developed. Computational tomography uses advanced gratings to produce overlapping projections of spectral cubes on 2D sensors. Then, using a complex algorithm related to the computed tomography (CT) reconstruction algorithm, the spectral cube is extracted from the overlapping projection data [27]. However, over-reliance on large-area sensors greatly limits the spatial and spectral resolution. The Hadamard transform uses a Hadama transform template instead of a slit [28,29], and each pixel measures multiple spectral channels superimposed on multiple target points in a single measurement, thus greatly increasing the luminous flux; however, the acquisition time is extended, the structure is complex and the spatial light modulator limits the resolution. Duke University’s Brady research group presented a direct view coded aperture snapshot spectral imager [24,30]. Their method undersamples the scene spatially and uses compressed sensing for reconstruction; however, the reconstruction relies on a complex algorithm, which depends on the trade-off between the spatial resolution of the signal and the image quality, replacing the trade-off between spatial resolution and spectral resolution. This means that the quality of the resulting data is unpredictable, and spatial and spectral reconstruction artifacts are usually introduced. Therefore, the obtained spatial resolution is reduced.

In summary, hyperspectral imaging remote sensing technology, including dispersive, filter-based and interferometric technologies, has matured and has been widely used in space remote sensing detection but is limited by the detection principle, making it difficult to improve the spatial resolution and detection efficiency significantly. For all kinds of new spectral imaging technologies, although they have the advantages of high throughput, the immature technology, the performance of the device and the quality of the acquired data, etc., along with the overall performance, mean that these novel approaches can not keep up with the traditional spectral imaging technology, which is difficult to use in space remote sensing practical applications.

Based on the above analysis, it can be seen that in the field of space optical remote sensing technology, luminous flux has been the central problem limiting the detection capability of instruments. In contrast, space hyperspectral remote sensing technology is limited by the imaging principle, and its detectors receive weaker light energy than panchromatic remote sensing cameras. The development of new high-throughput spectral imaging techniques, therefore, holds promise for breaking the bottleneck of the current hyperspectral remote sensing technology. This paper proposes a broadband filter-based spectral measurement method combined with panchromatic imaging remote sensing to form a novel high-throughput spectral imaging technique based on broadband spectral modulation and multi-channel combined measurement methods. The core idea of the method is to replace spectroscopic measurement with broadband spectral multi-channel combined measurement to obtain high luminous flux, which can significantly improve the application performance index and has high research and application value.

## 2. The Measurement Model of Broadband Filter Spectra

The basic principle of our proposed broadband filter-based spectral measurement method can be summarized as follows. The detector receives linearly superimposed light energy from multiple spectral bands. If multiple modulations and combined measurements of the broadband spectral range are made by the detector before it receives light energy, then it is possible to invert the spectral distribution of the incident spectrum through the inverse of the spectral modulation function; i.e., the detection of the target spectrum is completed. Described in the following mathematical model, the output signal of the photodetector is expressed as

(1) $$S=\int_{\lambda_{0}}^{\lambda_{1}}R\left(\lambda \right)E\left(\lambda \right)d\lambda ,$$

where *S* is the detector output signal, R(λ) is the spectral detector response, $\lambda_{0}$ is the lower bound of the system spectral response, $\lambda_{1}$ is the upper bound of the spectral response, λ is the wavelength and E(λ) is the distribution of the incident spectral intensity with wavelength. Recasting Equation (1) as a discrete form gives us

(2) $$S=\sum_{\lambda_{0}}^{\lambda_{1}}R\left(\lambda \right)E\left(\lambda \right).$$

Modulating and measuring the incident spectrum *t* times can form a system of equations for measuring the incident spectrum:

(3) $$\left\{\begin{array}{c} S_{1}=\sum_{\lambda_{0}}^{\lambda_{1}}R_{1}\left(\lambda \right)E\left(\lambda \right)\\ S_{2}=\sum_{\lambda_{0}}^{\lambda_{1}}R_{2}\left(\lambda \right)E\left(\lambda \right)\\ \;\;\;......\\ S_{t}=\sum_{\lambda_{0}}^{\lambda_{1}}R_{t}\left(\lambda \right)E\left(\lambda \right) \end{array}\right..$$

which can be expressed as

(4) $$\left[S_{k}\right]=\left[R_{k}\left(\lambda \right)\right]\left[E\left(\lambda \right)\right],$$

where $\left[S_{k}\right]$ is a t×1 column matrix, where each element represents the *k*th measurement signal; $\left[R_{k}\left(\lambda \right)\right]$ is a t×t matrix, where the row direction represents a group of spectral modulation functions and the column direction represents the *k*th group of spectral modulation functions; and [E(λ)] is a t×1 column matrix, where each element represents the incident spectral irradiance corresponding to the wavelength of the spectral modulation function.

**Figure 2:**
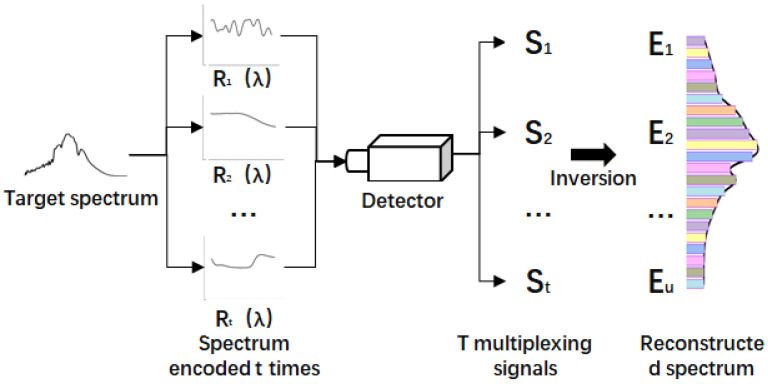
Spectral reconstruction process.

The process of spectral reconstruction is the process of solving Equation (4). The spectral reconstruction is completed by solving [E(λ)]. The measurement principle is shown in Figure 2.

**Figure 3:**
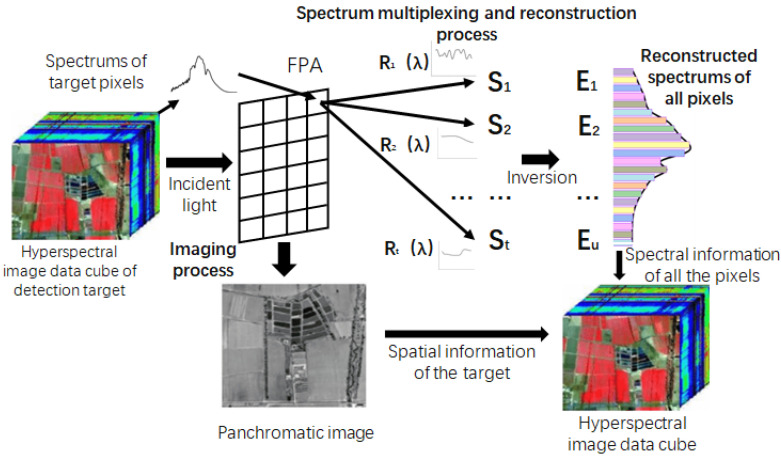
Broad-spectrum multi-channel spectral imaging principle.

The above process is for only one pixel on the detector, and if the above process is extended to every pixel on the detector and the same operation described above is repeated for each pixel, the data cube for the spectral imaging of the target scene can be inverted to obtain the target scene. The principle of spectral imaging based on spectral dimensional multi-channel measurements is shown in Figure 3.

We use a mathematical model to illustrate the principles of building spectral imaging based on spectral dimensional multi-channel measurements. Assume that the number of pixels of the detector is P×Q, extending Formula (1) to two dimensions, we obtain

(5) $$S_{mn}=\int_{\lambda_{0}}^{\lambda_{1}}R_{mn}\left(\lambda \right)E_{mn}\left(\lambda \right)d\lambda ,$$

where $S_{mn}$ is the output signal of the *n*th column of the *m*th row of the detector array, $R_{mn}\left(\lambda \right)$ is the spectral response of the nth column of the mth row of the detector array and $E_{mn}\left(\lambda \right)$ is the spectral irradiance incident on the surface of the detector pixel. Recasting Equation (5) as a discrete form gives us

(6) $$S_{mn}=\sum_{\lambda_{0}}^{\lambda_{1}}R_{mn}\left(\lambda \right)E_{mn}\left(\lambda \right).$$

By performing broadband spectral imaging for the target with a CMOS (complementary metal oxide semiconductor), the CMOS detector will output the signals captured by each pixel to form a signal matrix $\left[S_{mn}\right]$ to form a gray-scale image, which demonstrates the accomplishment of the acquisition of spatial information for the spectral imaging process.

Then, we modulate the spectral response $R_{mn}\left(\lambda \right)$ of each pixel to obtain *t* spectral responses $\left[R_{mn}\left(\lambda \right)\right]$ and then use the modulated spectral response $R_{mnt}\left(\lambda \right)$ to image the same target *t* times; thus, we can obtain

(7) $$\left[S_{mnk}\right]=\left[R_{mnk}\left(\lambda \right)\right]\left[E_{mn}\left(\lambda \right)\right],$$

where $\left[S_{mnk}\right]$ is a t×1 column matrix, where each element represents the *k*th measured signal of the *m*th row and the *n*th column of the pixel; $\left[R_{mnk}\left(\lambda \right)\right]$ is a t×t matrix, where the row direction represents a certain set of modulated spectral responses of the pixel, and the column direction represents the *k*th set of modulated spectral responses; and $\left[E_{mn}\left(\lambda \right)\right]$ is a t×1 column matrix, where each element represents the incident spectral irradiance of the pixel.

We measure $\left[S_{mnk}\right]$ from the output signal of the sensor array, and by precisely calibrating all modulated spectral responses $\left[R_{mnk}\left(\lambda \right)\right]$ of all pixels of the sensor array, we can reverse the distribution of the incident spectral energy of each pixel with wavelength $\left[E_{mn}\left(\lambda \right)\right]$. Combined with the ordinary imaging process represented by Equation (6), we can finally obtain the spectral imaging data cube of the target scene in the spectral range of $\lambda_{0}$ to $\lambda_{1}$.

This method of multi-channel measurement in the spectral dimension does not deal with the spatial dimension of the incident signal. However, it only requires the modulation of the incident optical signal in the spectral dimension and combined measurements. For detectors for everyday use, the spectral response of the pixels does not differ greatly and does not reach the modulation of the spectral dimensional incident signal. The simplest way to change the spectral response of the system is to use filters with different spectral transmittances; thus, broadband filters were chosen to modulate the spectral response.

The process of spectral reconstruction is the process of solving Equation (7). The accuracy of the reconstructed spectra is influenced by the calibration uncertainty of the spectral response $\left[R_{mnk}\left(\lambda \right)\right]$ and the uncertainty caused by the inversion algorithm. This reconstruction of the spectrum is only sufficiently accurate and reliable if Equation (7) has a well-posed solution. Equation (7) shows an appropriate solution to the problem of dealing with the pathological nature of the coefficient matrix $\left[R_{mnk}\left(\lambda \right)\right]$ of Equation (7). In order to ensure that Equation (7) has a well-posed solution, the number of measurements must be greater than or equal to the number of spectral channels. The accuracy of the solution is improved by constructing a superdetermined set of equations using more modulations and measurements. Thus, a Tikhonov-based regularization algorithm was used to solve this overdetermined pathological equation in order to invert the spectrum accurately.

## 3. Tikhonov Regularization Algorithm

Measurement errors and noise are inevitable, and due to these two errors, Equation (7) is severely pathological, resulting in a difference between the actual spectrum and the recovered reconstructed spectrum. To reduce this difference, the Tikhonov algorithm is chosen to solve this pathological equation, and the optimal solution is obtained using the Tikhonov algorithm. Tikhonov regularization is a fall-back approximation used to solve inverse problems that are impossible or difficult to solve exactly and which are thus made solvable within the bounded allowable error by imposing a constraint.

For the linear system (7), we solve the inverse problem: the monitoring data *S* and the response matrix *R* are known, and we must find the solution for the source term *E*. *R* is the pathological matrix, and the value of *E* is unusually sensitive to *S*. Therefore, it is not possible to find a direct solution for S=R×E to obtain *E*, and so we introduce the Tikhonov regularization method to solve this ill-posed problem and obtain a relatively real spectrum *E*.

Below, we illustrate mathematically the regularization method used in this paper. For ease of reading, we will equate the equation S=R×E into the linear algebraic form: Ax=b (*A* corresponds to the detector spectral response *R*, x corresponds to the spectral intensity *E* and b corresponds to the detector output signal *S*).

Our idea is to define the regularized solution $x_{\lambda }$ as the minimizer of the following weighted combination of the residual norm and the side constraint:

(8) $$x_{\lambda }=\mathrm{argmin}\left\{{∥Ax-b∥}_{2}^{2},+,\lambda^{2},{∥L\left(x,-,x^{*}\right)∥}_{2}^{2}\right\}.$$

where *A* is the coefficient matrix and right-hand side b, $x^{*}$ is an initial estimate, the matrix *L* is typically either the identity matrix $I_{n}$ or a p×n discrete approximation of the (n−p)-th derivative operator, in which case *L* is a banded matrix with full row rank, and the regularization parameter λ controls the weight given to minimization of the side constraint relative to the minimization of the residual norm.

The parameter λ also controls the sensitivity of the regularized solution $x_{\lambda }$ to perturbations in *A* and b, and the perturbation bound is proportional to $\lambda^{-1}$. Thus, the regularization parameter λ is a necessary quantity that controls the properties of the regularized solution, and λ should, therefore, be chosen with care.

We use generalized cross-validation (GCV) to select the regularization parameters λ. GCV is based on the philosophy that if an arbitrary element $b_{i}$ of the right-hand side b is left out, then the corresponding regularized solution should predict this observation well, and the choice of regularization parameters should be independent of an orthogonal transformation of b.

This leads to the choice of the regularization parameter that minimizes the GCV function.

(9) $$G\equiv \frac{{∥Ax_{\mathrm{reg}}-b∥}_{2}^{2}}{{\left(\mathrm{trace}(I_{m}-AA^{I})\right)}^{2}},$$

where $A^{I}$ is a matrix which produces the regularized solution $x_{\mathrm{reg}}$ when multiplied with b; i.e., $x_{\mathrm{reg}}=A^{I}b$. The filter factors can be used to evaluate the denominator by means of the simple expression

(10) $$\mathrm{trace}\left(I_{m}-AA^{I}\right)=m-\left(n-p\right)-\sum_{i=1}^{p}f_{i}.$$

where the numbers $f_{i}$ are filter factors for the particular regularization method. For Tikhonov regularization, which plays a central role in regularization theory, the filter factors are either $f_{i}=\sigma_{i}^{2}/\left(\sigma_{i}^{2}+\lambda^{2}\right)\left(\;\mathrm{for}\;L=I_{n}\right)$ or $f_{i}=\gamma_{i}^{2}/\left(\gamma_{i}^{2}+\lambda^{2}\right)\left(\;\mathrm{for}\;L\neq I_{n}\right).$

In [31], it is illustrated that the GCV method seeks to balance perturbation and regularization errors. We finally decided to adopt the quasi-optimality criterion for our selection.This method is only defined for a continuous regularization parameter λ and amounts to minimizing the function.

(11) $$Q\equiv \lambda {∥\frac{dx_{\lambda }}{d\lambda }∥}_{2}={\left(\sum_{i=1}^{p}{\left(f_{i}\left(1-f_{i}\right)\frac{u_{i}^{T}b}{\gamma_{i}}\right)}^{2}\right)}^{1/2}.$$

The numbers $\sigma_{i}$ are the singular values of *A*, and the vector $u_{i}$ is the left singular vector of *A*. We defined the ratios as

(12) $$\gamma_{i}=\sigma_{i}/\mu_{i}.$$

As demonstrated in [31], under certain assumptions, the approach also corresponds to finding a good balance between perturbation and regularization errors in $x_{\lambda }$.

## 4. Experimental Validation

In order to verify the feasibility of modulation of the incident spectrum by a broadband filter to recover the spectrum of the detected target and the influence of various factors, we used the three-dimensional spectral data cube (PaviaU) of a feature obtained from an existing hyperspectral imager as the simulated incident spectrum, reconstructed the spectrum using the algorithm established in Section 3 and then evaluated the accuracy or distortion of the reconstructed spectrum.

We used 98 broadband filters with a spectral range of 430–860 nm. The general laboratory calibrated the detector’s spectral response *R*, and the uncertainty was able to reach about 2%; that is, the standard deviation was 1% in the experiment. We used this error level as input. The light intensity data of the spectrum through the broadband filter was obtained using the Equation (7), and the spectrum was reconstructed using the algorithm in Section 3.

### 4.1. Selection of Indicators to Evaluate the Effectiveness of Reconstruction

In order to analyze the effectiveness of the broad-spectrum imaging system for reconstructing the spatial and spectral information of the target image and the accuracy of the reconstructed spectra, we used a hyperspectral imager which acquired a three-dimensional data cube of the target object as the incident light spectrum in a simulation experiment with a spectral interval of 4.2 nm. The spatial sample size was 610 × 340 × 103 pixels. The GCV-based Tikhonov algorithm was used to reconstruct all the pixels to obtain the reconstructed data cube, and we randomly selected the single-wavelength image of a certain band to evaluate the spectral accuracy and image quality of the single-wavelength image.

For spectral accuracy, we used the mean squared error (MSE) and relativistic spectral quadratic (RQE) as evaluation criteria. The mean squared error is the expectation of the square of the difference between the estimated value of the parameter and the real value of the parameter. Mathematical expressions such as MSE (13) can evaluate the degree of variation in the data: the smaller the MSE, the better the model’s ability to fit the experimental data.

(13) $$MSE=\frac{1}{N}\sum_{t=1}^{N}{\left({\mathrm{predicted}}_{t}-\mathrm{label}\right)}^{2}$$

We also used the relativistic spectral quadratic (RQE) to analyze the distortion of the spectra of characteristic features. The formula is as follows:

(14) $$\mathrm{RQE}=\frac{\sqrt{\sum {\left(\phi \left(\lambda \right)-\varphi \left(\lambda \right)\right)}^{2}}}{\sum \varphi (\lambda )}.$$

where ϕ(λ) is the original data and φ(λ) is the reconstruction data; the greater the RQE, the greater the distortion.

The image quality was evaluated in two ways: the peak signal-to-noise ratio (PSRN) and structural similarity (SSIM).

Given an image J and K of size m×n, the MSE is defined as

(15) $$MSE=\frac{1}{mn}\sum_{i=0}^{m-1}\sum_{j=0}^{n-1}{\left[J\left(i,j\right)-K\left(i,j\right)\right]}^{2}$$

PSNR (dB) is then defined as

(16) $$PSNR=10\cdot \log_{10}\left(\frac{MAX_{J}^{2}}{MSE}\right)$$

where $MAX_{J}^{2}$ is the maximum pixel value of image J.

SSIM is defined as

(17) $$\mathrm{SSIM}\left(x,y\right)=\frac{2\mu_{x}\mu_{y}+C_{1}}{\mu_{x}^{2}+\mu_{y}^{2}+C_{1}}\cdot \frac{2\delta_{xy}+C_{2}}{\delta_{x}^{2}+\delta_{y}^{2}+C_{2}}$$

where $\mu_{x}$,$\mu_{y}$ are all pixels of the image block, $\delta_{x}$ and $\delta_{y}$ are the standard deviation of image pixel values, $\delta_{xy}$ is the covariance between *x* and *y*, and $C_{1}$ and $C_{2}$ are constants to avoid system errors when the denominator is zero.

### 4.2. Results of Different Algorithms for Spectral Reconstruction

In this set of experiments, we used 98 broadband filters, to which we applied random noise which obeyed a normal distribution with a standard deviation of 1%, to recover 52 channels in the dataset PaviaU with a spectral range of 430–860 nm. We used Equation (7) to obtain the light intensity data S of the spectrum passing through the broadband filter, and the matrix conditional number forming *R* was $1.038\times 10^{5}$, forming part of the problem of solving serious ill-conditioned equations. To demonstrate the effectiveness of our algorithm for spectral reconstruction, we inverted the spectra E using the least-squares method (LSQR), the Tikhonov algorithm based on the L-curve method to select regularization parameters and the Tikhonov algorithm based on the GCV method to select regularization parameters, as listed in Section 3.

**Figure 4:**
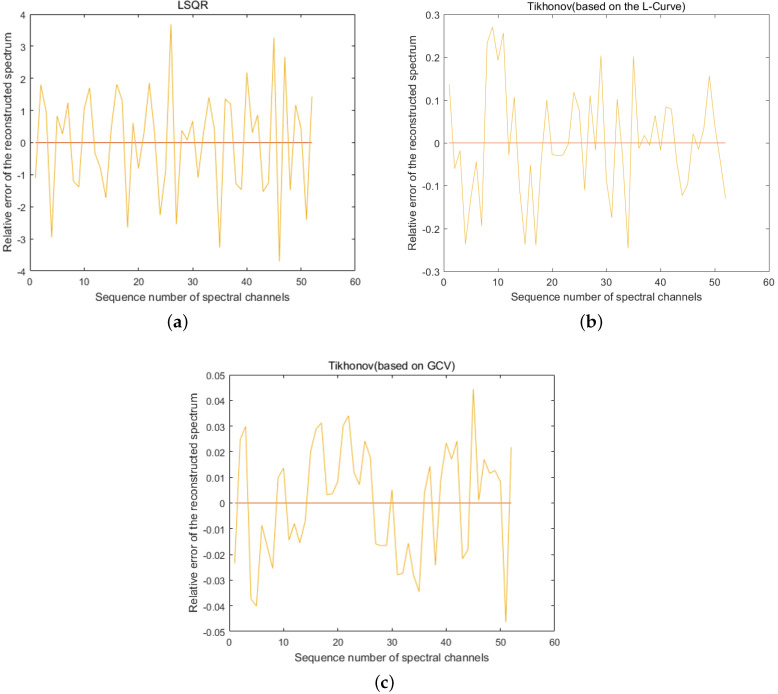
Reconstruction of the spectrum by the algorithm when the modulation function *R* calibrates the standard deviation σ to 1%. (**a**) The spectral accuracy recovered using the least squares (LSQR) method reached a maximum error of 400%. (**b**) The maximum error in spectral accuracy recovered using the Tikhonov method based on the L-curve reached 28%. (**c**) The maximum error in spectral accuracy recovered using the generalized cross-validation (GCV)-based Tikhonov method was only 5%.

**Figure 5:**
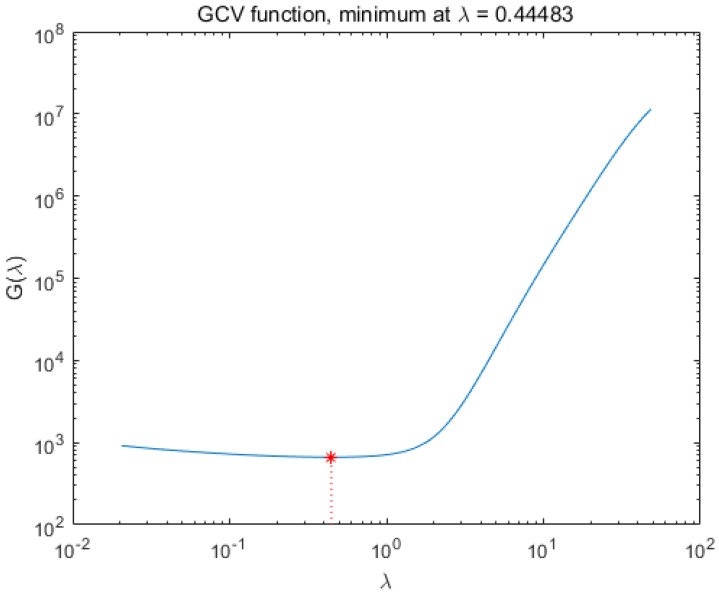
Finding the regularization parameter λ when recovering the best results using the GCV-based Tikhonov method.

From Figure 4, we can see that when the standard deviation σ of the modulation function *R* is 1%, the maximum error of the accuracy of LSQR for spectral reconstruction reaches 400% and the maximum error of the recovery of the spectrum based on the L-curve-based Tikhonov algorithm reaches 28%. However, we use the GCV method to find the regularization parameters of the Tikhonov algorithm. Figure 5 shows that when regularization parameter λ is 0.44483, the best recovery effect on the spectral recovery is exhibited. The maximum error of the spectral reconstruction effect does not exceed 5%.

**Figure 6:**
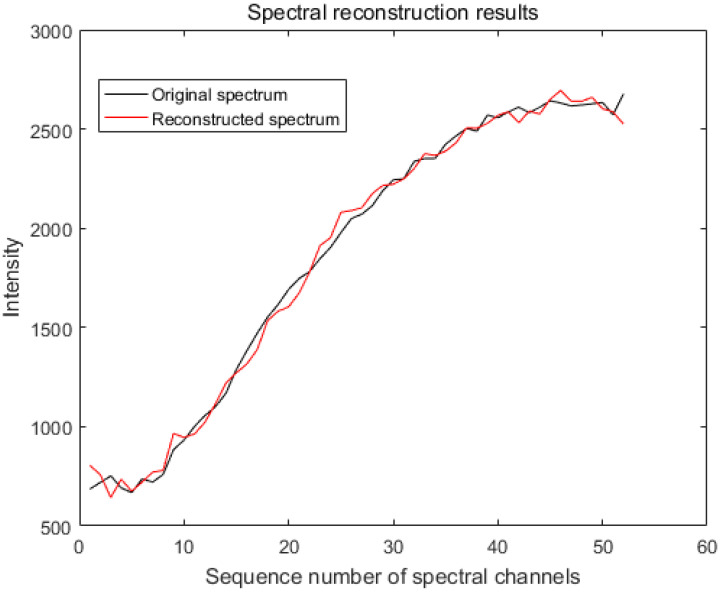
Original spectrum and reconstructed spectrum using the GCV-based Tikhonov algorithm (the mean squared error (MSE) is $6.8\times 10^{-4}$ and the relativistic spectral quadratic (RQE) is 0.0278).

We measured the accuracy of the reconstructed spectra using the evaluation criteria in Section 4.1. In the simulation, we took a point in the dataset PaviaU (coordinates 305,170) and normalized the intensity of the spectra. The spectra were evaluated using the LSQR, L-curve-based Tikhonov and GVD-based Tikhonov algorithms, respectively. The MSE of the spectrum after LSQR reconstruction was $1.5\times 10^{7}$ with an RQE of 70.7; based on the L-curve, the spectral MSE after the Tikhonov reconstruction was $4.0\times 10^{6}$ with an RQE of 52.5; and based on the GVD, the spectral MSE after Tikhonov reconstruction was $6.8\times 10^{-4}$ with an RQE of 0.0278 (Figure 6).

**Figure 7:**
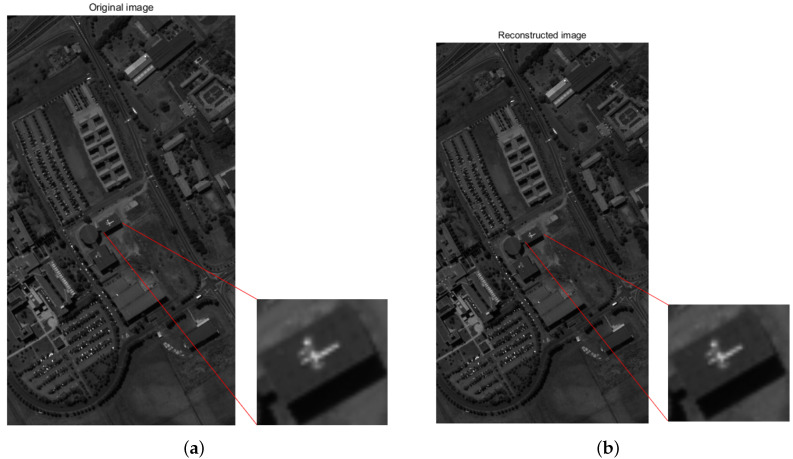
When the modulation function *R* calibrates the standard deviation σ to 1%, the algorithm is used to reconstruct the data cube PaviaU for restoration. The spectra of 52 channels were recovered using 98 broadband filters, and one of the single-wavelength images was taken to compare the recovery effect. (**a**) shows the original single-wavelength image, and (**b**) shows the recovered single-wavelength image. Local detail images are shown enlarged. (peak signal to noise ratio (PSNR): 40.1974; structural similarity (SSIM): 0.9885).

The MSE of the reconstructed single wavelength image was 6.2136, and the PSNR was 40.1974. The SSIM was 0.9885. Figure 7 shows that our algorithm can recover not only the spectrum but also the spectral image accurately, which illustrates that high-precision spectral imaging data reconstruction is possible.

### 4.3. Effect of Algorithm Stability and Other Factors on Accuracy

To avoid accidental interference with the data, we measured the stability of our algorithm using the Monte Carlo method, and we conducted 1000 independent experiments on the GCV-based Tikhonov algorithm. When the modulation function *R* calibration standard deviation σ was 1%, the maximum recovery error did not exceed 4.8% when using our algorithm to reconstruct the spectrum.

**Figure 8:**
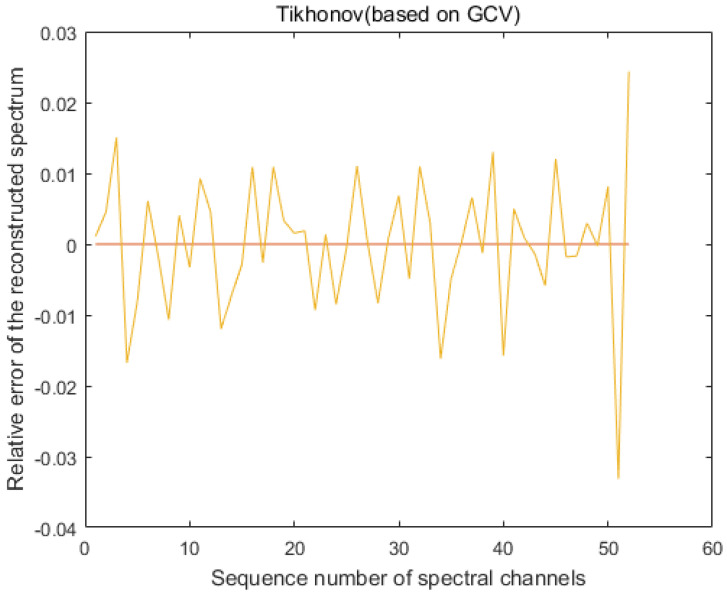
Recovery of the mean error of spectra using the GCV-based Tikhonov algorithm for 1000 independent experiments using the Monte Carlo method.

**Figure 9:**
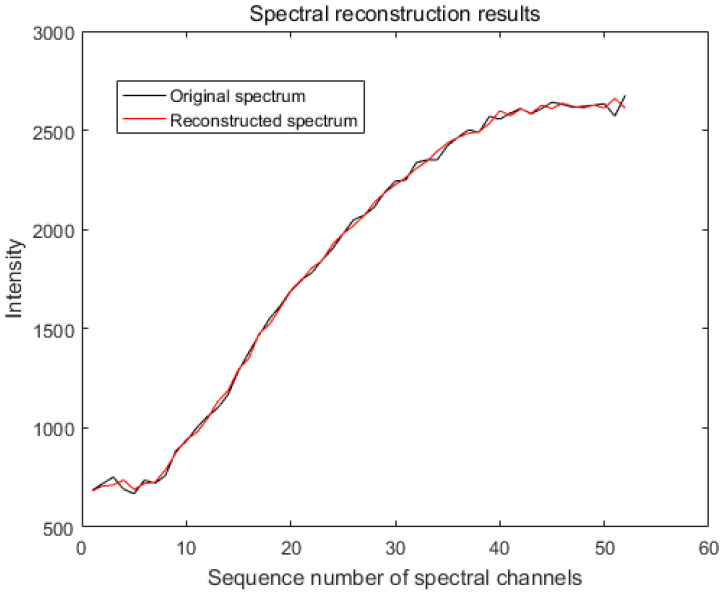
Spectra recovered from 1000 independent experiments using the Monte Carlo method using the GCV-based Tikhonov algorithm vs. the original spectra.

We obtained the average error result of 1000 iterations, as shown in Figure 8. Compared with Figure 4c, we found that when we performed the Monte Carlo experiment, the average error result was better than the maximum error of one experiment. The spectrum is shown in Figure 9, indicating that our algorithm has good stability.

**Figure 10:**
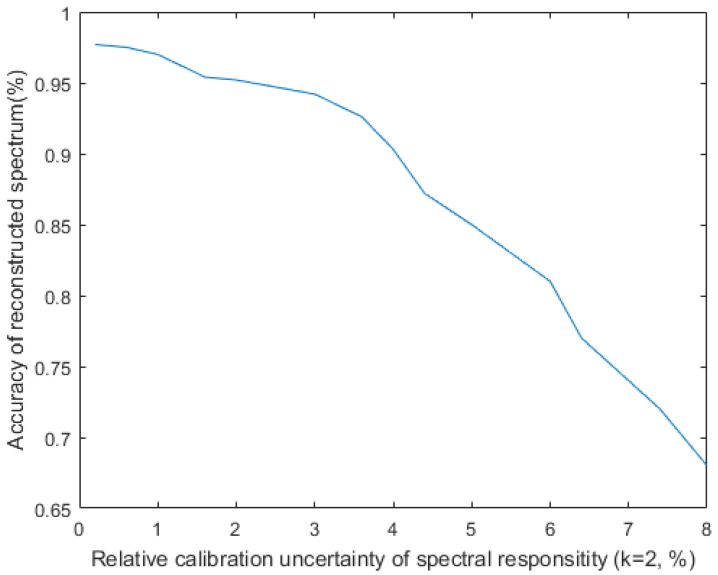
Effect of the calibration uncertainty σ of *R* on the accuracy of reconstructed spectra.

We assigned different standard deviations σ to the modulation function *R*.The Monte Carlo method was then used to reconstruct the spectrum (1000 independent experiments) and take the maximum error to draw a curve. The results are shown in Figure 10. We can see that when the error of R is 4%, the spectrum reconstruction accuracy is less than 90%, and when the error of R is less than 2%, the spectrum recovery accuracy is about 95%.

**Figure 11:**
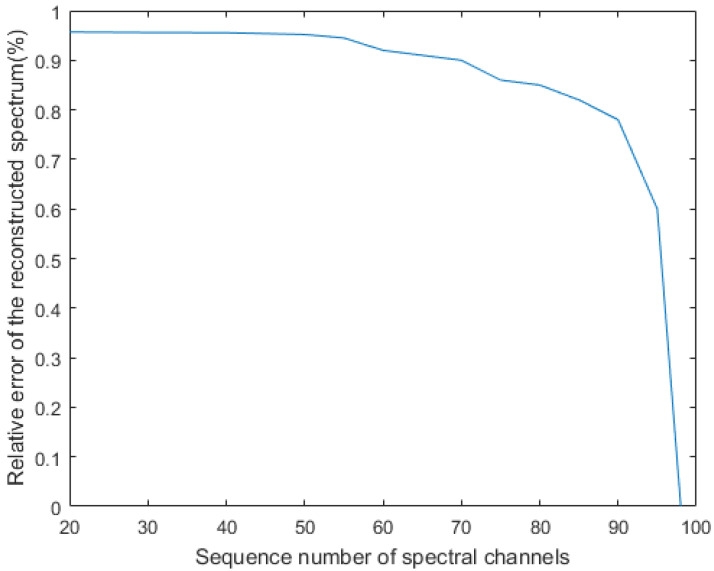
Effect of the number of equations on the accuracy of spectral reconstruction.

We also consider the effect of the number of overdetermined equations on the accuracy of spectral recovery. We used 98 broadband filters to reconstruct the spectra with different numbers of channels, and the error of the detector spectral response *R* was 2%; the experimental results are shown in Figure 11. When we recovered the spectra of 52 channels or fewer, the spectral accuracy of the reconstruction was able to reach 95%. We observed that when 98 broadband filters were used to recover the spectra of 98 channels, the recovered spectral accuracy was close to 0, indicating that under this definite favorable situation, the effect of reconstructing the spectrum is very poor. When there is an error in the detector spectral response *R*, the conditions of the overdetermined equation must be satisfied to better reconstruct the spectrum.

**Figure 12:**
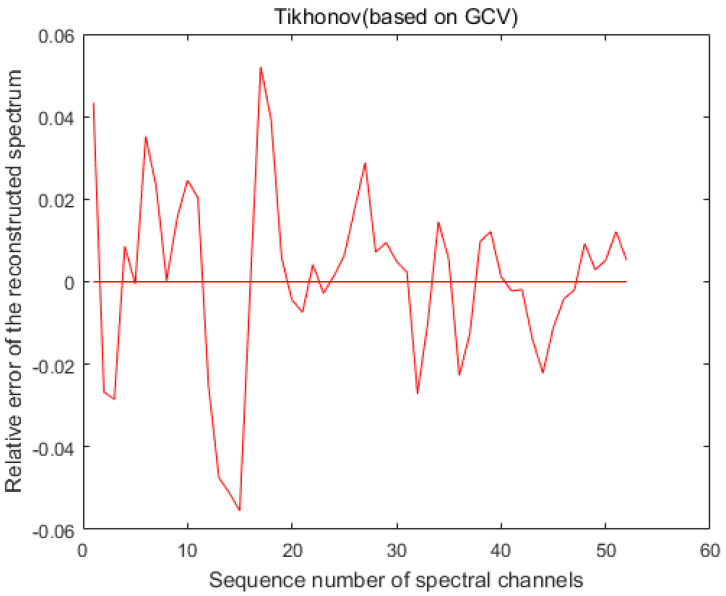
Maximum error in the reconstructed spectrum considering a detector SNR of 40 dB.

When the noise of the detector was considered and the signal-to-noise ratio was 40 dB, we attempted to reconstruct the spectrum; the spectral recovery results are shown in Figure 12. When the SNR was 40 dB, the maximum error in reconstructing the spectra was around 5%.

## 5. Conclusions

We used measurement modulation to achieve spectral reconstruction, using a broadband filter to modulate the incident spectrum and then inverting the spectrum with the signal acquired by the detector with a known spectral modulation function. We used the GCV-based Tikhonov regularization algorithm to restore the spectrum. We recovered the spectra using a GCV-based Tikhonov regularization algorithm when the standard deviation σ of the modulation function *R* was 1%; the maximum error of the restoration result of the spectrum was 4.8%. Furthermore, as a comparison, we reconstructed the spectra using the least-squares and L-curve based Tikhonov algorithm, which showed that our algorithm recovered the spectra with higher accuracy with the same error. To demonstrate the stability of the algorithm, we conducted 1000 independent experiments using the Monte Carlo method. The results of 1000 independent experiments indicated that the maximum error did not exceed 4.8%. In addition, we took one of the single wavelength images of the recovered data cube and performed a quality evaluation with a peak signal to noise ratio of 40.1974 and a structural similarity of 0.9885. We then analyzed the effect of the error of the modulation function on the accuracy of the reconstructed spectra, and the spectral modulation parameters were calibrated with an uncertainty of 2% or less, and the reconstructed spectra could reach 95% accuracy. The effect of the number of overdetermined equations on the accuracy of the reconstructed spectra was likewise analyzed, quantifying the modulation of the incident spectrum using 98 broadband filters. We found that the spectral accuracy of the reconstruction could reach 95% for spectral channel counts below 52. Finally, the effect of detector noise on the results was analyzed, and our algorithm still showed good recovery when the detector noise was below 40 dB.

In this paper, we used a broadband filter as a spectral encoder to spectrally modulate the incident light and performed multiplexed measurements. We performed the spectral encoding and multiplexed measurements in a very simple manner, thereby greatly increasing the throughput of remote sensing. The concrete imaging mode of the instrument based on our theory can be designed as a multiple pushbroom camera or a staring camera, which are respectively suitable for different tasks.The approach proposed in this paper reduces the use of complex and expensive equipment and controls volume and cost, which is important for aerospace applications.
